# Acute glomerulonephritis with large confluent IgA-dominant deposits associated with liver cirrhosis

**DOI:** 10.1371/journal.pone.0193274

**Published:** 2018-04-10

**Authors:** Jessica Hemminger, Vidya Arole, Isabelle Ayoub, Sergey V. Brodsky, Tibor Nadasdy, Anjali A. Satoskar

**Affiliations:** 1 Department of Pathology, Division of Renal and Transplant Pathology, The Ohio State University Wexner Medical Center, Columbus, Ohio, United States of America; 2 Department of Internal Medicine, Division of Nephrology, The Ohio State University Wexner Medical Center, Columbus, Ohio, United States of America; Instituto Nacional de Ciencias Medicas y Nutricion Salvador Zubiran, MEXICO

## Abstract

**Background:**

Small glomerular IgA deposits have been reported in patients with liver cirrhosis, mainly as an incidental finding in autopsy studies. We recently encountered nine cirrhotic patients who presented with acute proliferative glomerulonephritis with unusually large, exuberant glomerular immune complex deposits, in the absence of systemic lupus erythematosus (SLE) or monoclonal gammopathy-related kidney disease. Deposits were typically IgA dominant/codominant. Our aim was to further elucidate the etiology, diagnostic pitfalls, and clinical outcomes.

**Methods:**

We present clinical features and kidney biopsy findings of nine cirrhotic patients with an unusual acute immune complex glomerulonephritis. We also identified native kidney biopsies from all patients with liver cirrhosis at our institution over a 13-year period (January 2004 to December 2016) to evaluate presence of glomerular IgA deposits in them (n = 118).

**Results:**

Six of nine cirrhotic patients with the large immune deposits had a recent/concurrent acute bacterial infection, prompting a diagnosis of infection-associated glomerulonephritis and treatment with antibiotics. In the remaining three patients, no infection was identified and corticosteroids were initiated. Three of nine patients recovered kidney function (one recovered kidney function after liver transplant); three patients developed chronic kidney disease but remained off dialysis; two patients became dialysis-dependent and one patient developed sepsis and expired shortly after biopsy. Within the total cohort of 118 patients with cirrhosis, 67 others also showed IgA deposits, albeit small; and 42 patients had no IgA deposits.

**Conclusions:**

These cases provide support to the theory that liver dysfunction may compromise clearance of circulating immune complexes, enabling deposition in the kidney. At least in a subset of cirrhotic patients, a superimposed bacterial infection may serve as a “second-hit” and lead to acute glomerulonephritis with exuberant immune complex deposits. Therefore, a trial of antibiotics is recommended and caution is advised before immunosuppressive treatment is offered. Unfortunately, most of these patients have advanced liver failure; therefore both diagnosis and management remain a challenge.

## Introduction

Immune complex-mediated glomerulonephritis is characterized by deposits of polyclonal immunoglobulin (usually with complement) forming antigen-antibody complexes in the glomeruli with resultant kidney dysfunction [1]. Commonly encountered immune complex-mediated glomerulonephritides include IgA nephropathy, lupus nephritis (glomerulonephritis associated with systemic lupus erythematosus [SLE]), infection-associated glomerulonephritis (secondary to ongoing systemic infection, most commonly Staphylococcal infections), and post-infectious glomerulonephritis (occurring after resolution of infection, typically with Group A β-hemolytic Streptococcus). Optimal treatment targets the underlying etiology, and treatment can differ drastically depending on the cause. For instance, autoimmune disorders are treated with immunosuppression while infections require antimicrobial therapy and in fact immunosuppression could potentially lead to sepsis and even death.

While there are overlapping histologic, immunofluorescence, and ultrastructural features between the different etiologies of immune complex-mediated glomerulonephritis, the type and location of the glomerular immune complex deposits in conjunction with clinical history and laboratory findings usually point to the underlying etiology. For example, both IgA nephropathy and Staphylococcal infection-associated glomerulonephritis (SAGN) typically have IgA and C3 deposits and may have similar histologic findings, thus, can be difficult to distinguish based on biopsy findings alone. However, clinical history of a recent or ongoing systemic infection, recent onset hematuria and proteinuria would favor the latter [2–5]. Lupus nephritis typically shows large abundant IgG-dominant immune complex deposits involving the mesangium and/or glomerular capillary loops. It usually shows other immunoreactants as well leading to a “full-house” staining pattern [6]. IgA nephropathy characteristically has fewer and smaller IgA-dominant deposits that are usually paramesangial in location (close apposition to the glomerular basement membrane overlying the mesangium) [7]. Infection-associated glomerulonephritis also tends to have IgA-dominant, small, mesangial and/or subendothelial immune complex deposits, and in a small percentage of biopsies subepithelial “humps” may be seen [2]. Post-infectious glomerulonephritis typically shows C3-dominant deposits commonly forming subepithelial humps [5]. Thus, immunofluorescence and ultrastructural features play an important role in proper classification and treatment of glomerulonephritides.

Although infrequent, one can find mild, incidental IgA immune complex deposits in glomeruli that do not directly result in renal dysfunction/acute glomerulonephritis. One of the most common scenarios is mild IgA deposits seen in the setting of liver cirrhosis [7, 8] presumably because of the inability of the diseased liver to clear IgA-containing immune complexes from blood circulation. In contrast to this, we recently encountered nine patients with liver cirrhosis who presented with acute proliferative glomerulonephritis with large, exuberant immune complex deposits that stained for IgA and typically also IgG and C3. These patients had acute kidney injury with nephritic urine sediment and nephrotic range proteinuria. Although the large immune complex deposits and proliferative glomerular lesions were suggestive of active proliferative lupus nephritis, none of the patients had a history of SLE before or after the biopsy. Interestingly, six of the nine patients had a recent/concurrent bacterial infection. Thus, we hypothesize that a bacterial infection in the setting of advanced liver dysfunction can trigger excessive immune complex deposition in the glomeruli, characterized by unusually large IgA-containing immune-type deposits and resultant acute glomerulonephritis.

The aim of this study is to present the kidney biopsy findings, demographic features, and clinical outcomes of the nine cirrhotic patients with acute kidney injury and unusually large IgA-containing glomerular immune complex deposits. Additionally, we identified and reviewed kidney biopsies from all cirrhotic patients seen at our institution over a 13-year period to further characterize IgA deposits in this group of patients.

## Methods

We describe nine patients with liver cirrhosis who presented with acute kidney injury and an active immune complex-mediated glomerulonephritis with unusually large, exuberant immune complex deposits that typically showed IgA-dominant or codominant staining. Six of these nine patients presented to the Nephrology Division at the Ohio State University Wexner Medical Center (OSUWMC). Three of the nine patients presented at other hospitals, but their kidney biopsies were sent to our Renal Pathology Division at OSUWMC for diagnostic work-up.

Additionally, from our Pathology database we identified all native kidney biopsies from patients with cirrhosis over a 13-year period (January 2004 to December 2016). There were a total of 118 kidney biopsies from patients with liver cirrhosis. Besides the nine cases described above, there were 67 biopsies that had IgA-containing immune complex deposits albeit small and of the usual type; and 42 biopsies with no IgA-containing deposits. Demographic features, etiology of cirrhosis, and the extent of the IgA deposits in all these patients, are described.

All renal biopsies were processed using standard techniques for light microscopy, direct immunofluorescence, and electron microscopy. The intensity of immunofluorescent staining was scored as negative (0), mild (1+), moderate (2+), and strong (3+). We scored staining as trace if the intensity is between 0 and 1+ and/or if staining was very scant in distribution. The study was approved by the Office of Responsible Research Practice, Ohio State University Wexner Medical Center, IRB 2011H0364 prior to the beginning of the study. Patient consent was waived by the IRB committee. Additional reseach data has been provided in the Supplemental files.

## Results

The clinical and laboratory findings of the nine patients with liver cirrhosis and large confluent, IgA-containing immune complex deposits are described in Table 1. They presented with acute renal failure due to active glomerulonephritis, requiring temporary dialysis. Six patients had a documented recent/concurrent bacterial infection (patients 1–6 in Table 1) and received antibiotics. Of these six patients with infection, Patient 1 subsequently died from methicillin-resistant *Staphylococcus aureus* (MRSA) sepsis, patients 2 and 3 recovered partial kidney function after antibiotics, Patient 5 recovered kidney function after a liver transplant, and patients 4 and 6 remained dialysis-dependent. Three of the nine patients had a negative infectious disease work-up (patients 7–9 in Table 1) and were treated with corticosteroids. Patients 7 and 8 developed chronic renal disease, with persistent hematuria and proteinuria and died of unknown causes one and five months post-biopsy, respectively. Patient 9 slowly recovered renal function, but had persistent hematuria and proteinuria. None of the 9 patients had detectable monoclonal gammopathy on serum and urine immunofixation or serum free light chain predominance. The liver function tests and immune serology results in the nine patients are shown in Table 2.

**Table 1:** Demographic, clinical and laboratory features of the nine patients with cirrhosis whose biopsy showed large immune complex deposits.

Patient	Age (Yrs)	Race	Gen-der	Cause of Liver Cirrhosis	Other Co-morbidities	Site of Infection	Culture Result	S. cr. at time of biopsy (mg/dl)	Proteinuria	Hematuria	C3, C4 (mg/dl)	Purpuric Rash	Treatment	Follow-up
1	58	C	M	Cryptogenic cirrhosis (possibly PSC)	Crohn's disease, HTN	SBP, sepsis	Propionobacte-rium; MRSA pneumonia	7.7	6.5 g/24 hr	>20 rbcs/hpf	47,9 (low)	Absent	Vancomycin, Zosyn.	Expired within one month, dialysis-dependent, decompensated liver failure and MRSA sepsis.
2	68	C	F	NASH cirrhosis, anti-smooth muscle antibody positive	Atrial fibrillation, DVT, HTN, CAD	Cellulitis followed by UTI	Staphylococcus aureus and Enterococcus faecalis	6.2	23 g/24 hr;	>20 rbcs/hpf	109,22	Absent	Cephalexin for UTI	3 month followup by local nephrologist. S.cr. 3.1 mg/dl. No urinalysis results. Developed chronic kidney disease but off-dialysis.
3	48	C	M	Hep C-associated cirrhosis, Cryoglobulin test positive, RF negative	Intravenous drug use	Right arm abscess	Gram positive cocci, not further characterized	5.8	100 mg/dL	20–25 rbc/hpf gross hematur-ia	Undetectable	Absent	Vancomycin, Zosyn.	**Recovered** within one month, S.cr. 1 mg/dl. No proteinuria or hematuria at followup.
4	57	C	M	Hep B, Hep C & Alcoholic cirrhosis. Cryoglobulin test negative, RF negative.	Intravenous drug use	UTI	Klebsiella oxytoca and Serratia marcescens	4.4	U P/C 10.7	10–19 rbc/hpf	53, <8 (low)	Absent	Ciprofloxacin	12 days later s.cr. 3.0. Dialysis dependent. Pulmonary hypertension. Pt refused treatment and left against medical advice.
5	48	C	M	Alcoholic cirrhosis, RF 28 (0–20 IU/ml)	HTN	Leg cellulitis	Ggram negative bacilli, not further characterized	3.5	U P/C 2.3	1–4 RBC/hpf	61 (low), 12	Absent	Clindamycin, Zosyn, Vancomycin	One month following the biopsy, patient received liver transplant. At one year followup, s.cr. 0.9 mg/dl. No hematuria or proteinuria.
6	50	C	M	Hepatitis C and alcoholic cirrhosis; RF negative	Obesity	Pneumonia	No organisms identified	3.1	38 g/24 hrs;	1–4 rbc/hpf	49 (low), 16	Absent	Vancomycin, Zosyn	Developed ESRD, hepatocellular carcinoma. Received liver and kidney transplant one year after biopsy.
7	64	C	M	Cryptogenic cirrhosis, NASH, RF 78 (0–20 IU/ml)	Obesity,DM	None identified	Negative	5	0.4 g/24 hrs	>50 rbc/hpf)	45 (low), 16	Present	Prednisone	S.cr. 2.4 after one month of biopsy, chronic kidney disease. Persistent hematuria >50 /hpf. Urine protein 415 mg/24 hr. Expired in one month,cause unclear.
8	50	His	M	Alcoholic cirrhosis	Obesity,DM	None identified	Negative	5	100 mg/dL	40 rbc/hpf)	80,16	Absent	Prednisone	S.cr 1.5 to 1.6 mg/dl after three months. Urine protein 100mg/dl, blood 6–20 RBCs. Developed chronic kidney disease. Expired in 5 months, cause unclear.
9	43	C	M	Alcoholic cirrhosis, anti-centromere antibody	Obesity	None identified	Negative	2.7	12 g/24hr	>20 rbcs/hpf	79,16	Absent	Prednisone	S.cr. 2.0 mg/dl at 1 month, 1.2 mg/dl at 5 months, >20RBCs/hpf, 100 mg/dl protein.

PSC = primary sclerosing cholangitis; MRSA = methicillin-resistant Staphylococcus aureus; U P/C = urine protein/creatinine ratio; UTI = urinary tract infection; DM = diabetes mellitus; Hep B = hepatitis B; Hep C = hepatitis C; NASH = non-alcoholic steatohepatitis; SBP = sponaneous bacterial peritonitis. RF = rheumatoid factor; ESRD = end-stage liver disease.

**Table 2:** Liver function tests and autoimmune serology results in the nine patients with liver cirrhosis and large glomerular immune complex deposits. (normal reference values are shown in parenthesis).

Patient	Total Bilirubin (<1.5 mg/dl)	Direct Bilirubin (<0.3 mg/dl)	ALT (10–52 U/L); AST (14–40 U/L)	Alk Phos (32 to 126 U/L)	Alb (3.5 to 5 g/dl)	Blood ammonia (11–35 micromol/L)	PT (11–14 seconds)	INR (0.9–1.1)	PTT (24–34 seconds)	Autoimmune serology
1	3.8 at biopsy; one month later 34.8	1.4 at biopsy; one month later 21.9	10,25	158	1.6	not performed	18.9	1.6	41	Negative ANA, dsDNA, Sm-RNP, ANCA, RF, anti-mitochondrial Ab.
2	0.9	0.3	13,27	84	2.9	not performed	18.4	1.5	32	Negative ANA, ANCA, RF, Anti-smooth muscle antibody.
3	0.3	0.2	26;24	77	2.5	not performed	16	1.3	37.6	Negative ANA, dsDNA, Sm- RNP, ANCA, RF. Hep C PCR 1,000,000 IU/ml
4	0.8	0.2	8,22	48	4.5	not performed	18	1.5	37	Negative ANA, dsDNA, ANCA, RF, cryoglbulin test. Hep C PCR 2917 (IU/ml); Hep B 82493594 IU/ml
5	3	1.1	33,69	270	2.3	182	15.2	1.2	36	Negative ANA, dsDNA, ANCA, RF 28 (0–20 IU/ml).
6	1.8	0.8	26;69	53	2.8	81	17.1	1.4	38	Negative ANA, dsDNA, ANCA, RF. Hep C PCR 1425931 IU/ml
7	2.3	0.8	22;36	85	2.3	166	18.8	1.6	30	Negative ANA, dsDNA, ANCA, RF 78 (0–20 IU/ml).
8	1 at biopsy; four months later 2.5	not performed at biopsy; 4 months later 1.5	42;22	169	2.6	not performed; 4 months later 56	12.3	1.2	not performed	Negative ANA, dsDNA, Sm- RNP, ANCA, RF
9	2.8	1.4	27; 60	168	2.8	50	15.5	1.2	not performed	Negative ANA, dsDNA, RNP, anti-Scl, anti-mitochonfrial Ab, anti-smooth muscle Ab, ANCA, RF, only positive anti-centromere Ab.

Alk Phos = alkaline phosphatase; ALT = alanine aminotransferase; AST = aspartate aminotransferase;S. alb = serum albumin; PT = prothrombin time; PTT = partial thromboplastin time, anti-Scl = anti-scleroderma antibody.

**Fig 1:**
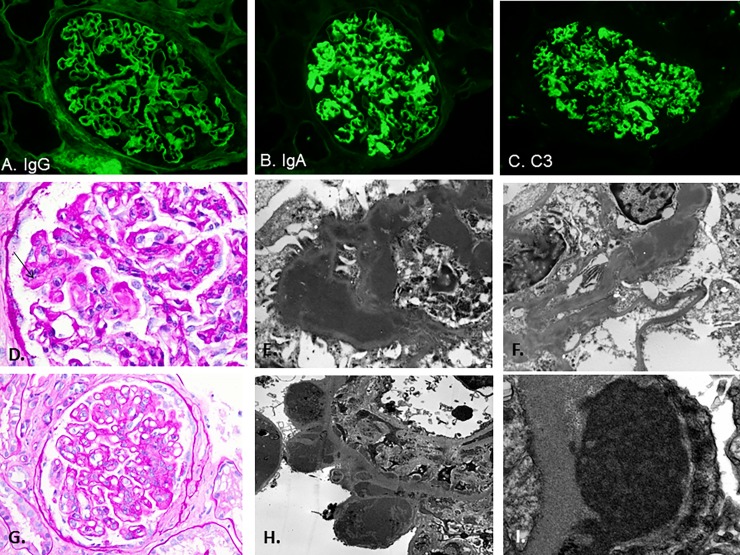
**Light microscopy, immunofluorescence, and electron microscopy findings: A-F, Patient 1 and G-I, Patient 6.** A) Mild staining for IgG (400x). B) Strong staining for IgA (400x). C) Strong staining for C3 (400x). D) Thickened capillary loops with "wire-loop lesions” (arrow) (Periodic Acid Schiff; 630x). E) and F) Large subendothelial electron dense immune-type deposits on ultrastructural examination (lead citrate and uranyl acetate fixation; 4,000x and 12,000x, respectively). G) Thickened capillary loops (Periodic Acid Schiff; 400x); H) and I). Large subepithelial electron dense immune-type deposits (lead citrate and uranyl acetate fixation; 6000x and 12,000x, respectively).

**Fig 2:**
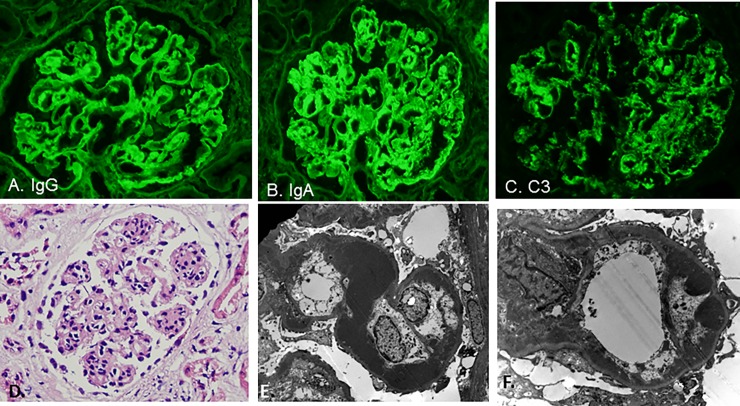
Light microscopy, immunofluorescence, and electron microscopy biopsy findings in patient 7. A) Strong mesangial and capillary loop staining for IgG (400x). B) Strong mesangial and capillary loop staining for IgA (400x). C) Strong mesangial and capillary loop staining for C3 (400x). D) Endocapillary hypercellularity (hematoxylin & eosin; 400x). E) and F) Large bulky mesangial and subendothelial electron dense immune-type deposits on ultrastructural examination (lead citrate and uranyl acetate fixation; 6,000x and 10,000x, respectively).

The histologic features seen in the renal biopsies of these nine patients are described in Table 3 and shown in Figs 1 and 2. Also see supplemental information (S1 Table). The main features on light microscopic examination include mesangial and endocapillary hypercellularity as well as segmental “wire-loop lesions” and occasional “hyaline thrombi” in the glomerular capillaries, indicative of abundant immune complex deposition. “Wire-loop lesion” is a term typically used in the context of proliferative lupus nephritis and refers to large immune complex deposits in the glomerular capillary walls that impart a glassy, hypereosinophilic appearance on hematoxylin and eosin (H&E) stain [6]. “Hyaline thrombi” is a term used to describe eosinophilic, PAS-positive globules that resemble hyaline seen within glomerular capillary lumens. “Hyaline thrombi” are characteristic of intraluminal cryoglobulin precipitates but may also represent large immune complex deposits in the glomerular capillary wall that bulge into the capillary lumens and are occasional seen in lupus nephritis [6].

**Table 3:** Kidney biopsy findings in the nine cirrhotic patients with large immune complex deposits.

	Light Microscopy				Immunofluorescence							Electron Microscopy
Pt.	Glomerular lesions	ATN	RBC casts	IF/TA	IgG	IgA	C3	C1q	K	L	Distribution of IF staining	Distribution of the large immune-type deposits
**1**	**Mesangial and segmental endocapillary hypercellularity, wire loop lesions, rare necrotizing lesion, few hyaline thrombi**	**Present**	**Present**	**2+**	**1+**	**3+**	**3+**	**+/-**	**3+**	**2+**	**mesangial, segmental GCL**	**Mesangial, subendothelial**
**2**	**Mesangial and segmental endocapillary hypercellularity, rare crescent**	**Present**	**Present**	**1+**	**2–3+**	**2–3+**	**2+**	**1+**	**3+**	**1+**	**mesangial, segmental GCL**	**Mesangial, intramembranous, few subepithelial**
**3**	**Mesangial and global endocapillary hypercellularity, wire loop lesions, rare necrotizing lesion, & few hyaline thrombi.**	**Present**	**Absent**	**1+**	**1–2+**	**2–3+**	**2–3+**	**1+**	**1+**	**1+**	**mesangial, GCL, intraluminal**	**Mesangial, intramembranous, subendothelial**
**4**	**Mesangial and endocapillary hypercellularity. wire loop lesions**	**Present**	**Absent**	**1+**	**3+**	**1+**	**3+**	**3+**	**3+**	**3+**	**mesangial, GCL**	**Mesangial, intramembranous, subendothelial, subepithelial**
**5**	**Mesangial and segmental endocapillary hypercellularity, wire loop lesions**	**Present**	**Rare**	**1+**	**2+**	**2–3+**	**2–3+**	**+/-**	**1+**	**3+**	**mesangial, segmental GCL**	**Mesangial, subendothelial**
**6**	**Mesangial and segmental endocapillary hypercellularity, wire loop lesions, & few small hyaline thrombi.**	**Present**	**Absent**	**2+**	**1–3+**	**3+**	**2–3+**	**1–3+**	**3+**	**3+**	**Mesangial, diffuse GCL**	**Mesangial, intramembranous, subendothelial, few subepithelial humps, TRIs**
**7**	**Mesangial hypercellularity**	**Present**	**Absent**	**1+**	**3+**	**3+**	**3+**	**+/-**	**3+**	**2+**	**Mesangial, segmental GCL**	**Mesangial, subendothelial**
**8**	**Mesangial and segmental endocapillary hypercellularity, wire loop lesions**	**Present**	**Present**	**1+**	**2+**	**2–3+**	**2+**	**Absent**	**3+**	**3+**	**Mesangial, diffuse GCL**	**Mesangial, intramembranous, subendothelial, subepithelial**
**9**	**Mesangial and segmental endocapillary hypercellularity, wire loop lesions**	**Present**	**Present**	**1+**	**+/-**	**3+**	**2–3+**	**Absent**	**2+**	**2+**	**Mesangial, segmental GCL**	**Mesangial, intramembranous, subendothelial**

GCL = glomerular capillary loops; Immunofluorescence staining intensity: +/- = trace; 1+ = mild; 2+ = moderate, 3+ = strong; Interstitial fibrosis and tubular atrophy: 1+ = less than 25%, 2+ = 25–50%, 3+ = greater than 50%; TRIs = endothelial tubuloreticular inclusions; IF/TA = Interstitial fibrosis and tubular atrophy; K = kappa light chain;:-Lambda light chain.

Immunofluorescence and ultrastructural findings in these biopsies demonstrated abundant immune complex deposition. Immunofluorescence showed moderate to strong staining for IgA and C3 with at least moderate IgG in eight cases. One case (patient 4) had IgG and C3 codominant staining with mild IgA. Predominance of one light chain was noted in biopsies of patients 2 and 5, but neither was completely negative and also serum and urine protein electrophoresis and immunofixation workup was negative. No monoclonal protein was detected in any of these patients. Electron microscopy confirmed the presence of unusually large glomerular electron dense immune-type deposits that were primarily mesangial and subendothelial in distribution. Additionally large subepithelial immune-type deposits (“humps”) were seen in one biopsy (patient 6). Microtubular or finger-print substructure was not present in the deposits.

Table 4 shows demographic information and causes of liver cirrhosis in the 118 patients with kidney biopsy. Among the 67 patients with cirrhosis and small usual-type IgA deposits, 40% were diabetic and among the 42 remaining patients with cirrhosis but no renal IgA deposits, 33% were diabetic, but only 1/9 patients with large immune deposits (patient 7) was known to be diabetic. Interestingly, 11/42 (26%) of the patients without IgA deposits had received a liver transplant in the past. Among the cirrhotic patients with small IgA deposits, only 2/67 (2.9%) had received prior liver transplant, and none among the nine patients with exuberant deposits.

**Table 4:** Cirrhotic patients with kidney biopsy, over a 13-year period (n = 118). Demographic features and etiology of liver cirrhosis.

Demographic features and etiology of liver cirrhosis	Biopsies with large exuberant IgA-containing immune complex deposits n = 9	Biopsies with small IgA deposits n = 67	Biopsies with no IgA deposits n = 42
Mean age	54 +/- 8.6	60 +/- 9.4	60.5 +/- 10.8
**Gender**	**8 M; 1 F**	**39 M; 28 F**	**25 M; 17 F**
**Ethnicity**	** **	** **	** **
**Caucasian**	**8**	**44**	**34**
**African American**	**0**	**10**	**7**
**Other**	**1 (Hispanic)**	**1 (Vietnamese)**	**1 (Hispanic)**
**Not known**	**0**	**12**	**0**
**Diabetes mellitus**	**1 (11%)**	**27 (40%)**	**14 (33%)**
**History of liver transplant prior to biopsy**	**0**	**2 (2.9%)**	**11 (26%)**
**Duration between liver transplant and renal biopsy**	**n/a**	**132 to 168 months**	**4 to 264 months**
**Causes of liver cirrhosis**	** **	** **	** **
**Hepatitis C**	**3**	**27**	**15**
**Hepatitis B**	**1**	**1**	**2**
**Hepatitis C and Hepatitis B**	**1**	**2**	**2**
**Alcoholic**	**3**	**11**	**8**
**Hepatitis C and alcoholic**	**2**	**2**	**0**
**Non-alcoholic steatohepatitis**	**1**	**12**	**0**
**Hemochromatosis**	**0**	**1**	**3**
**Primary biliary cirrhosis**	**0**	**2**	**3**
**Caroli**	**0**	**1**	**0**
**Cryptogenic**	**2**	**3**	**3**
**Autoimmune**	**0**	**1**	**0**
**idiopathic**	**0**	**4**	**7**

M = males; F = females

Table 5 shows the biopsies classified according to underlying kidney disease and the intensity of IgA staining in the biopsies of the 76 cirrhotic patients with glomerular IgA deposits and the 42 patients without IgA deposits. Also see supplemental information (S2 Table). The strongest mean IgA staining was seen in the 9 cases with large exuberant immune complex deposits, closely followed by primary IgA nephropathy. Mild to moderate intensity was seen in biopsies with culture-proven infection-associated glomerulonephritis (n = 12) and in biopsies that were suspicious for infection-associated glomerulonephritis although bacterial infection could not be confirmed clinically (n = 13). Of note, in these thirteen cases, infection-associated glomerulonephritis was favored over IgA nephropathy due to clinical findings indicative of an increased likelihood of infection such as an acute presentation with recent onset hematuria and proteinuria, absence of longstanding hematuria, older patient age, recent surgical procedure or trauma, presence of intravenous catheters, and intravenous drug abuse [2–5,9]. IgA staining was overall trace to mild in the biopsies with other renal disorders, including other immune complex-mediated glomerulonephritides, diabetic nephropathy, acute tubular necrosis, and focal segmental glomerulosclerosis, among others.

**Table 5:** Kidney biopsy diagnoses among the 118 patients with cirrhosis, with and without glomerular IgA deposits. Mean intensity of IgA staining in the 76 patients with IgA deposits are shown.

Biopsy diagnoses	n (%)	Mean IgA intensity
**Biopsies with large exuberant immune complex deposits**	**9(11.8%)**	**2.5**
**Primary IgA nephropathy**	**3 (3.9%)**	**2**
**Infection-associated glomerulonephritis. (Clinical and/or microbiological evidence of bacterial infection present).**	**12 (15.7%)**	**1.4**
**Suspicious for infection-associated glomerulonephritis morphologically but presence of bacterial infection could not be confirmed.**	**13 (17.1%)**	**1.7**
**Other immune complex glomerulonephritides with IgA staining**	**14 (18.4%)**	**1**
**Hepatitis C**	**9**	** **
**Lupus nephritis**	**2**	** **
**Fibrillary**	**1**	** **
**Membranous**	**2**	** **
**Diabetic nephropathy**	**12 (15.7%)**	**0.7**
**Other diseases (non-immune complex mediated) with IgA staining**	**13 (17.1%)**	**0.8**
**Minimal change disease**	**1**	** **
**TMA**	**1**	
**Drug-induced interstitial nephritis**	**2**	
**Lymphoproliferative disease**	**1**	
**Acute tubular necrosis**	**3**	
**FSGS**	**2**	
**Chronic reflux nephropathy**	**1**	
**Calcineurin inhibitor nephrotoxicity (liver transplant patient)**	**1**	
**ANCA associated glomerulonephritis**	**1**	
**Biopsies with absent IgA staining**	**42 (36%)**	**n/a**
**Diabetic nephropathy**	**8**	
**Hepatitis C related MPGN**	**8**	
**Fibrillary**	**1**	
**Membranous glomerulonephritis**	**1**	
**PGNMIGD**	**1**	
**Minimal Change disease**	**1**	
**Focal segmental glomerular sclerosis**	**2**	
**Acute tubular necrosis (ATN)**	**8**	
**Interstitial nephritis**	**1**	
**Chronic renal injury**	**9**	
**Non-specific mesangial expansion**	**2**

PGNMIGD = proliferative glomerulonephritis with monoclonal IgG deposits.

## Discussion

A number of systemic diseases have been reported to be associated with development of IgA deposits in the glomeruli, also termed as “secondary IgA nephropathy” [7,8]. These include hepatobiliary diseases (mainly cirrhosis); gastrointestinal diseases (celiac disease and inflammatory bowel disease); and rheumatologic diseases (ankylosing spondylitis, rheumatoid arthritis, psoriatic arthritis). Among these, liver cirrhosis is the most commonly described association [10–13]. In autopsy studies, up to 65% of patients with cirrhosis have been reported to show mild IgA deposits in the glomeruli (with or without C3) [12–15]. The staining was described as mild and incidental and not causing renal dysfunction. The glomeruli showed at most mild mesangial expansion with or without mesangial hypercellularity. Experimental studies have shown that the liver plays an important role in the clearance of circulating immune complexes (both IgG and IgA immune complexes), and a functional liver prevents deposition of immune complexes in tissues such as the kidney [16]. The human liver has two routes of IgA metabolism. The Kupffer cells, which constitute the mononuclear phagocyte system in the liver, are thought to be the main effector cells for uptake and degradation of circulating immune complexes. Animal experiments have shown that large preformed immune complex lattices are rapidly removed by the Kupffer cells of the liver [17–20]. In addition, the hepatocytes express an asialoglycoprotein receptor that binds IgA1 via asialylated O-linked terminal glycans, and the complex is then endocytosed and presented to the lysosomal apparatus for degradation [21,22].

Similar to prior autopsy studies [12–15], our study also shows that 64% (76/118) of biopsied cirrhotic patients have glomerular IgA deposition. However, as depicted in Table 5, in 37/76 (49%) biopsies, the IgA staining was more than just mild and was associated with a proliferative glomerulonephritis. Of these, 3 were primary IgA nephropathy, 25 were attributed to or suspicious for infection-associated glomerulonephritis, and 9 were the ones with numerous exuberant immune complex deposits (6 of these patients did have recent bacterial infection, 3 had negative infectious disease workup). Our study shows that in a substantial proportion of cirrhotic patients with glomerular IgA, these IgA deposits are not just mild and non-pathologic, but in fact are associated with renal dysfunction. Underlying bacterial infection is a frequent cause of the active glomerulonephritis. This association had not been clearly elucidated in previous autopsy studies [10, 23–25].

Additionally, we describe in detail nine patients with liver cirrhosis who in fact had unusually large exuberant, immune complex deposits and presented with acute kidney injury (due to acute immune complex-mediated proliferative glomerulonephritis). The immune complex deposits typically showed strong IgA staining and moderate to strong C3 and IgG staining. In six patients, there was a known recent/concurrent infection. Thus, it is possible that the underlying liver dysfunction in combination with an active bacterial infection resulted in exuberant deposition of immune complexes in the kidney and development of an acute glomerulonephritis. This hypothesis is particularly supported by the fact that two patients (patients 2 and 3 in Table 1) recovered kidney function after elimination of the infection with antibiotic treatment. Patient 5 also had complete recovery of renal function after antibiotic treatment and liver transplantation. Of note, a similar case of cirrhosis with glomerulonephritis that improved upon liver transplantation has been reported previously [26]. Interestingly, among the cirrhotic patients without IgA deposits in our study, 26% had already received a liver transplant in the past, which was not the case in the cirrhotic patients with IgA deposits, suggesting the importance of liver function in IgA immune complex clearance.

It is unclear why only a small subset of cirrhotic patients forms these unusually large immune complex deposits. None of the other cases of infection-associated glomerulonephritis in our cohort of 67 cirrhotic patients showed such unusually large immune complex deposits. Comparing these 9 patients with our previously reported 76 patients with culture proven SAGN [2], we did not encounter cases with such exuberant immune complex deposition before. Three patients in the SAGN series [2] did show cryoglobulin-like hyaline thrombi in a few glomerular capillary loops, but none of these three patients had cirrhosis [5, 27]. Additionally 24/76 (31%) of the biopsies in that series showed subepithelial humps, out of which 4 patients were Hepatitis C positive, but did not have documented cirrhosis[2]. Therefore, the exuberant immune complex deposits s in the biopsies of these nine patients described in the present study is a unique finding. Also the average intensity and prevalence of IgA staining is much higher in these 9 biopsies (shown in Table 5). Among the previously reported 78 culture-proven SAGN cases, 25% of the biopsies had trace to negative IgA and the average intensity if staining was 1.5 [2]. Another notable difference was that diabetes mellitus which is known to be a common morbidity in patients with SAGN [2, 3, 4], was seen in 41% in our published series and even higher in others. In contrast, only one of these nine cirrhotic patients with exuberant immune deposits was found to be diabetic.

An underlying infection was not identified in 3/9 cirrhotic patients with the large, immune complex deposits (patients 7, 8, and 9 in Table 1) despite thorough cultures and imaging studies. The etiology for the active immune complex-mediated glomerulonephritis in these cases therefore remains unclear. An infection-driven process is less likely, although such a possibility cannot be entirely excluded. Primary IgA nephropathy is another possibility, although such exuberant immune complex formation is also very unusual for primary IgA nephropathy. These three patients were treated with corticosteroids however, only one patient gradually recovered some kidney function despite persistent hematuria and mild proteinuria. Two of the three patients died of unclear causes 1 and 5 months post biopsy.

Such prominent glomerular immune complex deposits do raise the differential diagnosis of lupus nephritis. However, serologies and clinical findings were not supportive of an underlying autoimmune disorder in these patients. Furthermore, immune complex deposits seen in lupus nephritis are characteristically IgG-dominant. Of note, a recent report by Sise et al. described a similar “lupus-like” immune complex-mediated glomerulonephritis in three patients with cirrhosis and hepatitis C treated with oral interferon-free direct-acting antiviral agents [28] and Takada etal and colleagues described two patients with IgA nephropathy featuring similar wire loop-like deposits in two patients with alcoholic cirrhosis [29]. Similar to our nine cirrhotic patients, the kidney biopsies had prominent IgA staining within the deposits. Two of the three patients suffered from lethal infections after commencement of immunosuppression therapy, and it’s unclear if an occult infection may have been a contributing factor. The history and immunofluorescence results were not consistent with other glomerulonephritides like proliferative glomerulonephritis with monoclonal IgG deposits (PGNMIGD) or C3 glomerulonephritis either. Lastly, cryoglobulinemic glomerulonephritis could be considered in the differential diagnosis, particularly in the three patients with a known hepatitis C infection (patients 3, 4, and 6). However, there was no evidence of a purpuric rash, arthritis, or other signs of cryoglobulinemic vasculitis in these patients. Furthermore, serum testing for rheumatoid factor was negative, and microtubular substructure was not seen in the immune-type deposits on ultrastructural examination. These factors, in addition to prominent IgA staining (in patient 3 and 6), make cryoglobulinemic glomerulonephritis less likely.

The high mortality rate noted in our small cohort was largely related to decompensated liver cirrhosis and poor overall health rather than the glomerulonephritis alone [30]. These patients had advanced cirrhosis and associated co-morbidities, including ascites, spontaneous bacterial peritonitis, and/or hepatic encephalopathy. Some also had diabetes mellitus, obesity, and/or hypertension.

It is difficult to make any generalized treatment recommendations, because our cohort is small and clinical outcomes were variable. Treatment will have to be tailored for each individual patient depending upon the etiology of cirrhosis. Management will include appropriate treatment for the complications such as ascites, hepatic encephalopathy, malnutrition, coagulopathy and spontaneous bacterial peritonitis. For the immune complex glomerulonephritis, our personal recommendation is to also include antibiotics, at least initially, until the possibility of an infectious process can be thoroughly excluded. We recommend that immunosuppression is best avoided until an infectious process can be safely excluded and given with great caution even if culture results are negative. None of the nine patients developed biopsy-related bleeding complications.

In conclusion, this is an observational study based on kidney biopsy findings and detailed clinical and laboratory data and our aim is to draw attention to an unusual immune complex-mediated glomerulonephritis in a subset of cirrhotic patients. It is characterized by large, confluent immune complex deposits, quite different from the usual type of deposits seen in most cases of infection-associated glomerulonephritis. They contain IgA along with moderate to prominent C3 and IgG. It is plausible that in many cases, these are caused by a combination of poor immune complex clearance due to advanced liver dysfunction and a superimposed bacterial infection. Therefore, clinical work-up for an underlying infection is essential. Further experimental studies elucidating the mechanism of the exuberant immune complex formation in the setting of cirrhosis are needed.

## Supporting information

S1 Table (XLSX)

S2 Table (XLSX)
